# The Flux Operator

**DOI:** 10.12688/f1000research.147989.1

**Published:** 2024-03-21

**Authors:** Vanessa Sochat, Aldo Culquicondor, Antonio Ojea, Daniel Milroy

**Affiliations:** 1 Lawrence Livermore National Laboratory, Livermore, California, 94550, USA; 2 Google, Inc., Mountain View, California, 94040, USA

**Keywords:** high performance computing, converged computing, cloud computing, Kubernetes, batch workloads, workload manager

## Abstract

Converged computing is an emerging area of computing that brings together the best of both worlds for high performance computing (HPC) and cloud-native communities. The economic influence of cloud computing and the need for workflow portability, flexibility, and manageability are driving this emergence. Navigating the uncharted territory and building an effective space for both HPC and cloud require collaborative technological development and research. In this work, we focus on developing components for the converged workload manager, the central component of batch workflows running in any environment. From the cloud we base our work on Kubernetes, the de facto standard batch workload orchestrator. From HPC the orchestrator counterpart is Flux Framework, a fully hierarchical resource management and graph-based scheduler with a modular architecture that supports sophisticated scheduling and job management. Bringing these managers together consists of implementing Flux inside of Kubernetes, enabling hierarchical resource management and scheduling that scales without burdening the Kubernetes scheduler. This paper introduces the Flux Operator – an on-demand HPC workload manager deployed in Kubernetes. Our work describes design decisions, mapping components between environments, and experimental features. We perform experiments that compare application performance when deployed by the Flux Operator and the MPI Operator and present the results. Finally, we review remaining challenges and describe our vision of the future for improved technological innovation and collaboration through converged computing.

## 1. Introduction

Portability, manageability, and modularity of complex, heterogeneous workflows is becoming increasingly important for high performance computing (HPC). In particular, the need for workflows to be extended to cloud environments is a key component of collaboration across an increasingly diverse set of computational resources, and a likely solution for “green computing” to ensure energy efficiency and optimal usage of shared resources. ^1^ Other demands for flexibility of compute include the increasing use of internet of things “IoT” remote devices to conduct research, ^2^^,^^3^ an explosion of hardware available on cloud platforms, ^4^^,^^5^ and the dynamic addition of external resources. ^6^ A powerful demonstration of need also comes from a series of events organized by the European Commission to assemble experts for discussion on innovation in the “computing continuum,” citing a strong need for flexibility for distributed systems, green and dynamic technologies, and an emphasis on open source software. The discussion continues with workshops emphasizing the importance of shaping Europe’s digital future. Given this landscape, any entity involved in the business of scaled computing will fall behind if these technological needs are not prioritized. ^4^

In cloud computing communities, machine learning workloads are also becoming increasingly important, ^7^^–^^10^ and the cloud container orchestration technology Kubernetes is becoming the de facto standard for orchestration of these workflows following its success orchestrating microservices. As of June of 2023, the Kubernetes project had over 74,000 contributors, making it the second largest open source project ever after Linux, and the “most widely used container orchestration platform in existence” ( the CNCF project report). It is the chosen platform for orchestration at over 70% of Fortune 500 companies. In recent years, the growing need for supporting batch workflows has led to the creation of the Kubernetes batch working group. The group designs and implements application programming interfaces (APIs) to enable cloud-native batch workflows and jobs, and works to transition Kubernetes from primarily a stateless, service-oriented architecture to one that can support states and jobs. The first stable release of highly parallel Indexed Jobs marked an unofficial declaration of Kubernetes supporting what, at face value, looked like more traditional workflows from HPC. With this development, the needs of the cloud computing communities overlapped better with the needs of HPC than ever before.

Batch processing has a long history in HPC, and consequently the community has deep expertise. The need to embrace more cloud-like features results from changing workload demands. While a traditional HPC job is, e.g., a parallel simulation with tightly-coupled problem subdomains, modern workflows include the gamut from ensembles of simulations to artificial intelligence (AI) and services. A standard workflow is no longer a single job that writes to a shared filesystem, but rather an assortment of tasks that vary in their needs for hardware, storage, and running times. Modern workflows are typically specified via directed acyclic graphs (DAGs) that not only indicate direction or order of dependencies, but also utilization of different architectures, services, and virtualization technologies. To support these workflows the high performance computing community needs the portability, flexibility, and automation enabled by cloud, and the ability to span both applications and services.

It would be in the best interest of cloud communities to learn from and adopt the best technological innovations from HPC and vice versa. These common interests are a motivation for collaboration, and the time is right for the convergence of not just these communities but the technologies themselves. This collaboration, or the convincing of one community to engage with the other, is arguably more challenging than development work. When approaching those on the HPC side, discussions of cloud adoption focus on performance, costs ^11^^,^^12^ and security. ^13^ Approaching from the cloud side, there can be a lack of understanding of high performance computing technologies, and how they differ from or might potentially improve microservices. The concerns about performance, cost, and security of cloud can be addressed by the fact that cloud environments can be public, private, or both. A technology such as Kubernetes could be deployed on-premises. In the case of the second, the cloud computing community needs convincing that they too can benefit from the adoption of HPC technologies. Increasing performance and efficiency by using techniques from HPC, and providing better transparency of underlying resources by way of low-level performance analysis, can lower costs and time to completion. A solution that falls in the middle would likely bring together the best of both worlds, but could come with compromises such as paying a performance penalty for flexibility. Ideally, a converged approach would improve performance of cloud-native technologies through effective hardware use, with increased flexibility offsetting any potential performance compromises for running with a higher level of abstraction.

To facilitate collaboration between cloud and high performance computing communities and create effective converged environments, each communities’ needs must be determined and addressed. First, a solid demonstration is needed that there are benefits for both cloud and HPC to take on attributes of the other side. For HPC, this means more modularity, portability, and automation. For cloud, this means more performant workflows, efficient use of hardware, schedulers, and communication protocols that span networking, applications, and storage. Secondly, examples of technologies that can bring together the performance of HPC with the flexibility of clouds in a best-of-both-worlds environment must be prototyped. This vision, or the space of technologies that exists between cloud and HPC can be described with the term “converged computing”. ^14^^,^^15^ In a converged computing landscape of the future not only will technologies from traditionally disparate communities be brought together, but traditionally disparate communities will be united culturally to identify shared goals and foster deeper, more fruitful collaborations.

While many areas of work can be tackled, it was logical to start with a workflow manager analogous to Kubernetes in the high performance computing community, with the common use case of running simulations or machine learning tasks. The Flux Framework, a novel hierarchical framework for resource management and scheduling, provides abstractions that parallel those in Kubernetes, including modularity, well-defined developer and user interfaces, and an ability to integrate and manage different types of resources. ^16^ It stands out from other resource managers because of its ability to manage the exploding complexity of modern workflows described previously. To start with modularity, in the same way that several components are combined to create Kubernetes, components from Flux Framework are assembled together to manifest in a workload manager that is referred to simply as “Flux.” This modularity can mean, for example, that a component of Flux could be used in Kubernetes, or vice versa. For developer interfaces, arguably a core ingredient of convergence is having common programming languages or bindings. Kubernetes, as it was developed at Google, chose to use the Go programming language, also designed at Google. ^17^ Flux also provides a rich landscape of language bindings, one of which is Go. These shared interfaces and modularity make convergence possible.

Given the overlapping need to schedule jobs, the first work in this space was to integrate the Flux scheduler “Fluxion” as a scheduler plugin for Kubernetes called “Fluence”. ^15^ The rationale for this early work was that Flux could benefit Kubernetes in several ways. Firstly, Flux represents and schedules resources via directed graphs, which is notably different from the default Kubernetes scheduler that selects work for nodes based on a feasibility score. In fact, Flux was created to address significant limitations of traditional HPC resource managers and schedulers by facilitating workload portability, handling high throughput job requests, and employing sophisticated techniques for flexible and fine-grained task placement and binding. ^16^ Enabling the Flux scheduler in Kubernetes would bring this same graph-based and hierarchical resource-aware approach to Kubernetes, and this early work demonstrated exactly that – improved performance against the default Kubernetes scheduler. ^18^ More efficient scheduling was demonstrating by enabling MPI-based CORAL2 workloads to run and scale in Kubernetes that, by way of Fluence, avoided pathological resource mappings and resource starvation. ^14^ This work also demonstrated a valuable point – that the scheduling provided by a workload manager must be able to concretely meet the resource demands of a workflow, but to do so efficiently and effectively to maximally utilize a set of computational resources.

Aside from the technological benefits that might come from convergence of these two specific technologies for end users and developers, enabling Flux to run in the cloud would also provide benefits for cloud vendors attempting to attract a larger HPC customer base. Current products that target HPC researchers (e.g., AWS Batch, ParallelCluster, Google Batch, Azure CycleCloud) present a more familiar experience and may help transition users to the cloud. Other products that do not deliver a familiar command line interface would require an on-boarding process. The realization that workflows could be seamlessly portable by way of Flux, and Flux could serve as a vehicle for the workflow to move between cloud and HPC, inspired the next round of work discussed in this paper. By making the full Flux workflow manager, with all components assembled, available in Kubernetes, workflow specifications that work on HPC with Flux would also work in a cloud environment. For cloud vendors, the HPC user base could more easily make a smooth transition to using cloud too.

This paper introduces the Flux Operator, ^19^ the next step in work to explore integration of a traditional HPC scheduler within Kubernetes. The Flux Operator is a Kubernetes operator ^20^ that handles all the setup and configuration of a fully fledged Flux cluster inside of Kubernetes itself, allowing the user to efficiently bring up and down an entire HPC cluster for a scoped set of work that optimizes for important aspects of HPC. This paper first reviews the design and architecture of the Flux Operator ( Section 2), discussing Kubernetes abstractions for efficient networking and node setup. We then discuss how these design decisions impact essential needs such as workflows that use message passing interfaces (MPI), and experimental features like scaling and elasticity ( Section 4). Finally, experimental work demonstrates the Flux Operator enable superior performance for a well-known HPC application over the MPI Operator, the primary available option at the time for running MPI workflows ( Section 3). The paper concludes with discussion for anticipated future work, considerations for workflow design, and vision for the future ( Section 5).

## 2. Methods

### 2.1 Implementation

This section details the architecture of the Flux Operator, first describing the design and needs of Flux, and how those are satisfied in Kubernetes. From the standpoint of a software architect, the task of designing the Flux Operator could be approached as a problem of matching software design patterns. Knowing that Kubernetes provides a set of components and application programming interfaces, a key challenge was to assemble the components in a way that would deploy the full Flux Framework stack running inside of Kubernetes. An ideal design might aim to achieve the keystone properties of Kubernetes applications, including but not limited to fault tolerance, load balancing, and elasticity. Abstractions for storage, networking, and workloads could be selected for this design, and with a mindset of portability, meaning that the software components would be in containers themselves. The following sections refer to two roles – an operator developer, or someone that designs and implements the Flux Operator itself, and an operator user, an individual that installs the operator and uses it. These architecture sections start with an overview of Kubernetes Operators, and then describe each component of Flux, and a mapping from traditional bare metal solutions to abstractions in Kubernetes.

**2.1.1 Kubernetes operators**

While individual components such as pods or services could be individually implemented and created in the Kubernetes cluster, the advent of programmatic operators in 2016 has hugely simplified this process for the developer user. A Kubernetes operator serves as a controller for one or more Kubernetes objects, meaning that a developer can express all of the custom logic needed for an application of interest in code that is compiled and run in Kubernetes. The operator implements a custom resource whose behavior is dictated by a custom resource definition (CRD), a YAML file with a specification of variables for the controller to use. For the Flux Operator, this custom resource is called a “ MiniCluster”. The basic design of a controller is a loop, running a reconciliation process until a cluster reaches a desired state requested by a user via this custom resource definition YAML specification. This is a declarative model in that the operator user can specify high level constructs such as the cluster size and application to run, and they don’t need to know the details of setting up a Flux cluster, nor do they need to consider orchestration or update of components. This approach is advantageous in that it exposes only the amount of detail that is needed for some number of jobs, and the complexity that would require niche expertise is hidden.

**2.1.2 Flux Framework**

The Flux Framework is a novel, graph-based resource manager that is typically deployed on-premises at HPC centers. ^16^ It won an R&D 100 award in 2021, ^21^ and is slated to be the primary scheduler for the upcoming exascale-class system “ El Capitan” at Lawrence Livermore National Laboratory. It is called a framework because several projects combine together to form the resource manager known as Flux. A summary of these projects is described in Table 1, and the interested reader is directed to the learning guide for an in-depth overview.

**Table 1.** Flux Framework projects.

Project	Description
Flux-core	Core services
Flux-pmix	Flux shell plugin to bootstrap OpenMPI
Flux-sched	Fluxion graph-based scheduler
Flux-security	Security code
Flux-accounting	User bank and accounting

While Flux can be described in terms of its modules or components, for the work here it will described as it is seen in the space of Kubernetes abstractions.

**2.1.3 A Flux MiniCluster**

**The node** Flux is typically deployed across nodes on an HPC cluster, where each node can be thought of as an addressable compute or storage unit, and as having a unique network address. Moving into the space of cloud, the physicality of the server goes away, and instead the basis of a node is a virtual machine. However, while Kubernetes itself is deployed on nodes, a fundamental object is the pod – an abstract slice of a node that is tasked with some unit of work to run one or more containers, and allocated a particular set of resources. Since the Kubernetes scheduler is designed to assign more than one pod to a single node, the first task in defining this cluster was to ensure that there was a mapping of one pod per actual physical node. The reason for this mapping is due to Flux not being able to detect running on a partial node, which is a result of its use of the portable hardware locality ( hwloc) library to discover resources. Pod isolation in Kubernetes is based on a kernel technology, cgroups, which allows for resource management on the level of the kernel. However, this abstraction does not take hardware into account, and so regardless of a resource specification, hwloc will detect the resources of the entire node. In the context of two pods running on one node, despite any resource management done via cgroups, the library would erroneously discover the resources of the entire node twice. This means that Flux would detect double the resources that are actually available, and could schedule too much work on a single physical node that, to the resource manager, is seen as two separate nodes with identical resources. The 1:1 mapping of pods to nodes was originally achieved by way of a resource specification, a strategy that required the user to ask for just under the upper limit of CPU and memory offered by their cloud instance of choice. This strategy was later improved to not require knowledge of the instances by way of rules for pod affinity and anti-affinity. These are essentially constraints that tells the scheduler to ensure one pod per node, each with a hostname for Flux.

**The cluster** While it would be possible to deploy individual pods onto nodes, early Kubernetes offered further abstractions for sets of pods such as deployments and Stateful or Replica sets, and in 2015, an abstraction called a Job that was the first of its kind to approximate a traditional HPC job with the intention to run and complete. As of 2021, the batch working group introduced the indexed mode addition to Job where the same work could be done in parallel, expecting 1 to *N* completions. In that each node of a simple Flux cluster would be identical aside from subtle differences in startup, although other abstractions were considered, an indexed Job was ultimately chosen. The indexed Job is ideal in that it inherits needed features from the base Job such as having states, and adds an ability to create duplicates of the pods. To create pods it uses a batched approach, which is also advantageous to introduce an indexed ordering that ensures the bigger numbers are downscaled first. This allowed us to easily design the operator to use the index 0 pod as the lead broker, and any scaling up or down of the cluster ( Section 4.1) would never risk deleting the lead broker. Within this cluster context, given the assignment of one pod to one node, for the remainder of this paper the terms “node" and “pod" are used interchangeably as they are mapped to the same resources, memory and CPU.

**Networking** One of the foundational components of Flux Framework is the scalable tree-based overlay network, or “TBON” that connects the core modules for scheduling and resource management. The Flux TBON is a rooted tree that features leader and follower processes (brokers), each of which is typically mapped to one node. The leader expects follower brokers to connect to it. Mapping this design to the indexed Job, the role of lead broker can be assigned to index 0, and the follower brokers to indices 1 through N-1. Along with being easy to remember, this design decision allows pods to be created in order of their index with the lowest first, which is ideal to have the lead broker up earlier for the follower brokers to find. ZeroMQ ^22^ is used for the discovery of the node and to bootstrap the cluster. In this process, the follower brokers identify their place in the cluster by way of their rank in a shared system configuration file, and then connect to the lead broker on a specific port via transmission control protocol (tcp). If the lead broker is not up first the worker will wait to try connecting again, and by default the ZeroMQ library falls back to a tcp default retry timeout that increases exponentially. In practice this means delayed cluster startup times waiting for the follower brokers to retry. The scheduler and resource manager combined with this set of brokers that can communicate to run jobs is called a Flux instance. A Flux instance can be on the system level, meaning shared by many users, or owned by a single user. In both cases, the Flux instances handle user requests for submitting or otherwise interacting with jobs. The instance itself is hierarchical because it can spawn sub-instances whose resources are a subgraph of their parent.

The networking setup can give each pod a unique address that can be written into the Flux system configuration, and used to identify lead and follower brokers. For the first naive implementation, the Flux Operator created the pods, retrieved the IP addresses after creation from the Kubernetes API, and then added corresponding entries to the / etc/ hosts file for domain name system (DNS) resolution. Automated management of the hosts file proved to be a bad design because it required restarting all the pods to recreate a configuration file with known hostnames. Instead, a later design created a Headless Service for the MiniCluster, meaning that each pod could be given a label, a key value pair, that was known to the Headless Service, and discovering the labeled pod would add it to the network provided by the service. The Job controller then assigns a predictible hostname, and the Headless Service makes the name discoverable to DNS. This series of steps is essential for Flux to identify each broker, and is supported by way of unique pod names that are published as A records. This simplified the creation of the cluster, and allowed for networking readiness as soon as the service object was ready. Once this is done and the brokers have connected over TCP, further communication for the overlay network is done via ZeroMQ sockets. ^16^ However, for the cases of workflows that use a message passing interface (MPI), Flux has built-in modules for MPI and communication, meaning that Flux simply uses standard MPI libraries that can rely on sophisticated networking fabrics or other high speed interconnects. This reliance on cloud hardware has proven to be a challenge when deploying the Flux Operator to different cloud providers, and is a focus of collaborative work.

**Volume mounts** In terms of configuration, Flux requires a system configuration file, a ZeroMQ CURVE certificate used to encrypt communication, and a means to start the brokers. In a traditional HPC setup, this means could be using a systemd service, however in a Kubernetes environment with containers, it means a start command for the container with conditional logic for the lead vs. follower brokers. In Kubernetes, all of the above configuration can be achieved via volume mounts provided via ConfigMap objects. By way of mounting configuration files and other needed files to each pod container as read-only volumes, all nodes in the cluster have access to them. These are mounted at / etc/ flux and / flux_operator for configurations and the starting script, respectively, and the choice of a root path affords discoverability.

The curve certificate presented a bootstrapping design problem, as the standard way to generate it is via Flux itself (ZeroMQ is compiled within and exposed via the flux keygen command). However, this content was also required to exist for the read-only volume before starting the pod container. For the earliest design of the Flux Operator, a one-off certificate generator container was brought up that ran this key generation command, and the key was printed to the log to be retrieved by the operator. It could then be straightforward to write into the ConfigMap to be shared by the MiniCluster pods. In a later design, by way of collaboration with authors of this paper following Kubecon Amsterdam’23 ^23^ this bootstrapping problem was further improved by compiling ZeroMQ directly into the Flux Operator, and using cgo to interact with it directly to generate the certificate content for the ConfigMap inside the operator. This removed an entire step to generate the one-off pod and reduced startup latency, and is a beautiful example of how sharing ideas and collaboration can lead to improvements in design and functionality. The open source nature of these projects is key for enabling collaboration that can result in faster innovation. ^24^^,^^25^

**A Flux container image** The libraries and software needed for Flux must be built into a common Flux container that is replicated by the indexed Job. This container would need to support running configuration steps for Flux upon start, and additionally include any application of interest to be run by the user. This is a design flaw in that most containerized applications for HPC have not been built with Flux, and would need to be built again to install not only Flux, but required shared libraries. While the HPC community is attuned to building and optimizing components for different architectures, ideally a more modular, cloud-native solution would not require such a substantial time investment. Approaches need to be investigated that can achieve separation of application logic from Flux while not impacting performance, and further work is needed to investigate this. Once required software and configuration files are present, setup continues to create either a single-user or site-wide installation of Flux. The Flux Operator opts for a single-user design, and enables customization via variables exposed on the CRD. This customization includes (but is not limited to) archiving of data, creating multiple users, starting in an interactive mode, starting a RESTful application programming interface, or creating a custom batch job. The final component of the container is the “entrypoint” or the command that is run when the container is started. This varies between the lead and follower brokers, where the lead broker typically starts with a command to run a job, and the follower broker starts expecting to connect and receive work.

### 2.2 Operation

**Figure 1.**
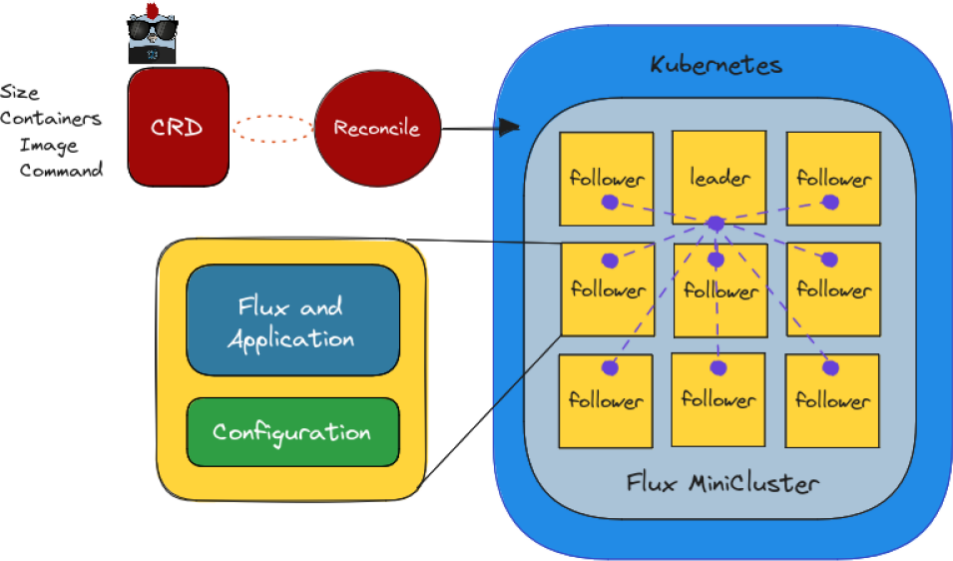
The Flux Operator design and creation process for a Flux MiniCluster. The custom resource definition “CRD” that describes the specification for the cluster is provided by the user as a YAML file. Upon creation, the operator reconciles in a loop until the state of objects in Kubernetes matches the desired state specified in the CRD. This desired state encompasses the creation of the MiniCluster, which includes an indexed Job with a group of pods for the cluster, each containing a Flux broker and volumes for configuration. A Headless Service (purple) networks the pods together to complete the MiniCluster.

The first requirement for using the Flux Operator is access to a Kubernetes cluster with sufficient permission to deploy each of the abstractions discussed in Section 2, including Deployment, Service, Job, and ConfigMap. The Flux Operator pre-built container images are provided for nodes with amd64 or arm64 architectures. Operation comes down to deploying these objects with the kubectl command line tool and then using the same tool to deploy the MiniCluster CRD. Upon creation, the operator reconciles in a loop until the state of objects in Kubernetes matches the desired state specified in the CRD. This desired state encompasses the creation of the MiniCluster, which includes an indexed Job with a group of pods for the cluster, each containing a flux broker and volumes for configuration. A Headless Service networks the pods together to complete the MiniCluster. The specific attributes of the cluster (e.g., size, application image, command) can be customized in this YAML file, resulting in a Flux MiniCluster that exists to run a command and clean up (akin to a batch job), or an interactive cluster that can address several use cases and interactions discussed in Section 4. The final Flux MiniCluster is illustrated in Figure 1.

## 3. Results

The Flux Operator is compared to another state-of-the-art Kubernetes Operator, the MPI Operator, which at the time of the experiments was considered the main option in the field for running MPI workloads. ^26^ The MPI Operator started as part of the Kubeflow project and defines an “ MPIJob” custom resource. Unlike the Flux Operator that coordinates between brokers with ZeroMQ, the MPI operator coordinates workers via secure shell (SSH). It requires an extra launcher node that serves the sole purpose of coordinating the workers, and akin to the Flux Operator, uses dedicated hostnames with an equivalent Headless Service. This launcher node conceptually could be thought of as analogous to the lead broker of the Flux instance in that it serves as an entrypoint for submitting jobs, however the main difference is that the Flux lead broker is considered part of the cluster to perform work. The MPI launcher node is not, and in practice this means the user will need to always incur the costs of an extra node just for the launcher. In practice, choosing a cheaper node for the launcher is a logical choice.

### 3.1 Experiment

**Figure 2.**
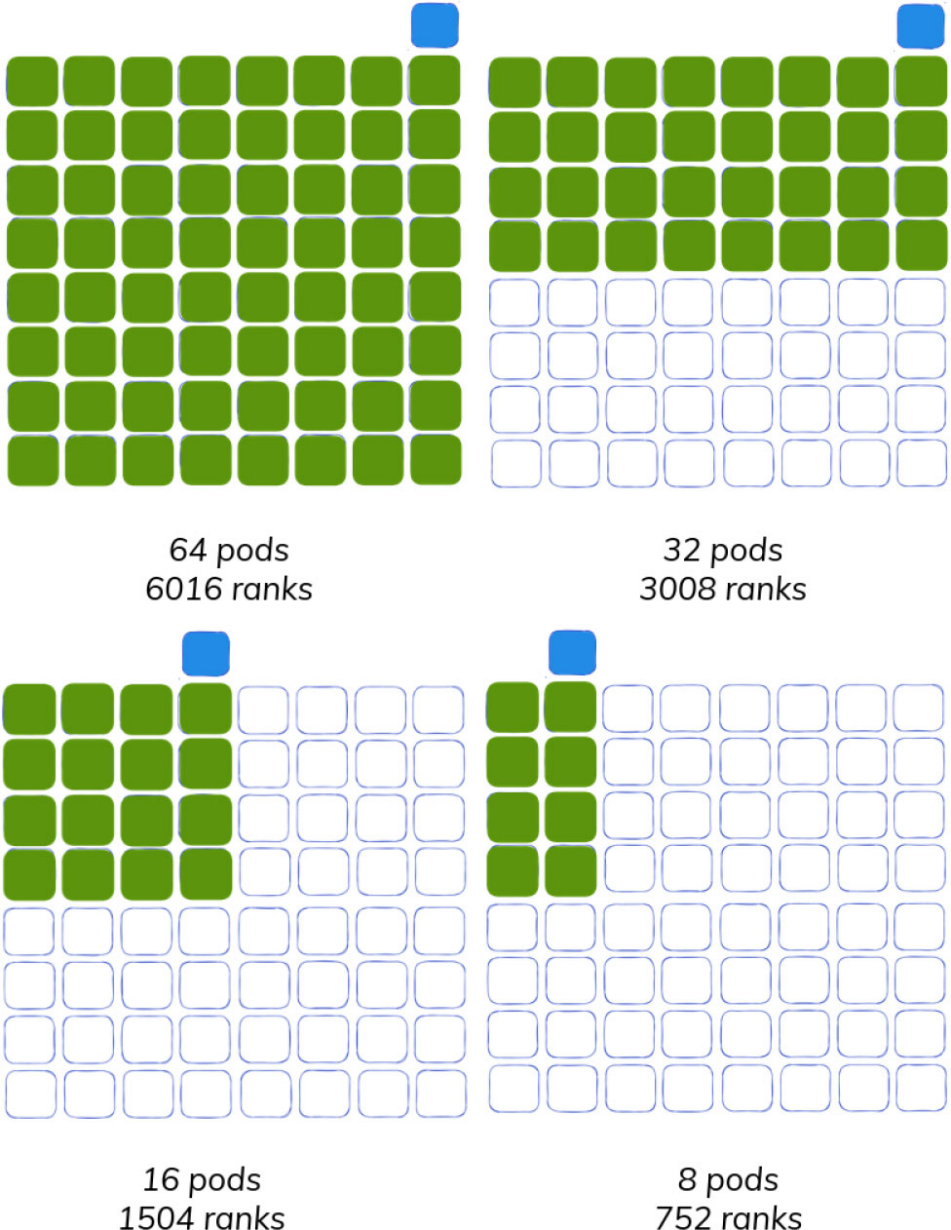
Experiment design for testing the Flux Operator against the MPI Operator. A single Kubernetes cluster of size 65 was created (blue outline) to test subsequently smaller cluster sizes, including 64 pods (6016 ranks), 32 pods (3008 ranks), 16 pods (1504 ranks), and 8 pods (752 ranks). An extra node (blue) is needed for the MPI Operator launcher to supplement the nodes doing work (green).

Experiments were conducted on Amazon Web Services Elastic Kubernetes Engine (EKS), using the hpc6a.48xlarge instance type hpc6a with the elastic fiber adapter (EFA) intending to test the Large-scale Atomic/Molecular Massively Parallel Simulator (LAMMPS) ^27^ in a strong-scaling configuration across cluster sizes and ranks of 64/6016, 32/3008, 16/1504, and 8/752, respectively ( Figure 2). This design was chosen to mirror previous work. ^18^ LAMMPS is used by the Collaboration of Oak Ridge, Argonne, and Livermore (CORAL) as a representative scalable science benchmark as part of CORAL-2. In testing, LAMMPS runs in parallel on MPI ranks (processes) across nodes, a molecular simulation ^28^ and the problem size 64×16×16 was chosen that would adequately test strong scalability across the chosen rank and node counts. LAMMPS scalability depends on network latency, and the experiment results report the total wall time recorded by the LAMMPS run as a metric for performance. A cluster setup that enables lower latency will run faster, and ideally the simulation should get faster as the number of nodes is increased with strong scaling. A second metric time of interest is the time for the launcher to submit and complete a job. For Flux this means timing the flux submit command that is given the command to run LAMMPS, and for the MPI Operator it means timing the mpirun command that does the same. The final metric time of interest was the total cluster creation and deletion times, which can be calculated based on the total runtime of the MiniCluster minus the LAMMPS total wall time. This time would include each pod preparing the broker, starting Flux, and networking with the lead broker. The runtime would ideally decrease across these chosen rank and node sizes.

To ensure the nodes of the cluster are consistent and do not influence results, experiments were run on the same Kubernetes cluster, and simply used smaller portions of it. As the MPI Operator requires an extra launcher node, the maximum cluster size needed (64) was increased by 1, resulting in a size 65 node cluster for these experiments. Finally, a modified version of the MPI Operator was used ^14^ to allow it to scale to over 100 MPI ranks.

The experiments proceeded as follows. The main Kubernetes cluster of size 65 is first created. Then, for each of the Flux Operator and MPI Operator:

1. Launch Job/create MiniCluster for size 64, 32, 16, 8
2. Run LAMMPS x 20
3. Record timings and save configuration files and logs

For each experiment run, a single “throwaway” run is first performed to pull the container with Flux and LAMMPS to the node, where it is cached for further runs. This ensures that time recorded in creating the MiniCluster does not include pulling the container image, which would have variability depending on the image size. The experiments are then run in an automated fashion using Flux Cloud, a simple experiment orchestration tool for running experiments with Flux MiniClusters on Kubernetes. All experiment code, configuration files, and tagged containers are available. ^29^^,^^30^

### 3.2 Figures of merit

**Figure 3.**
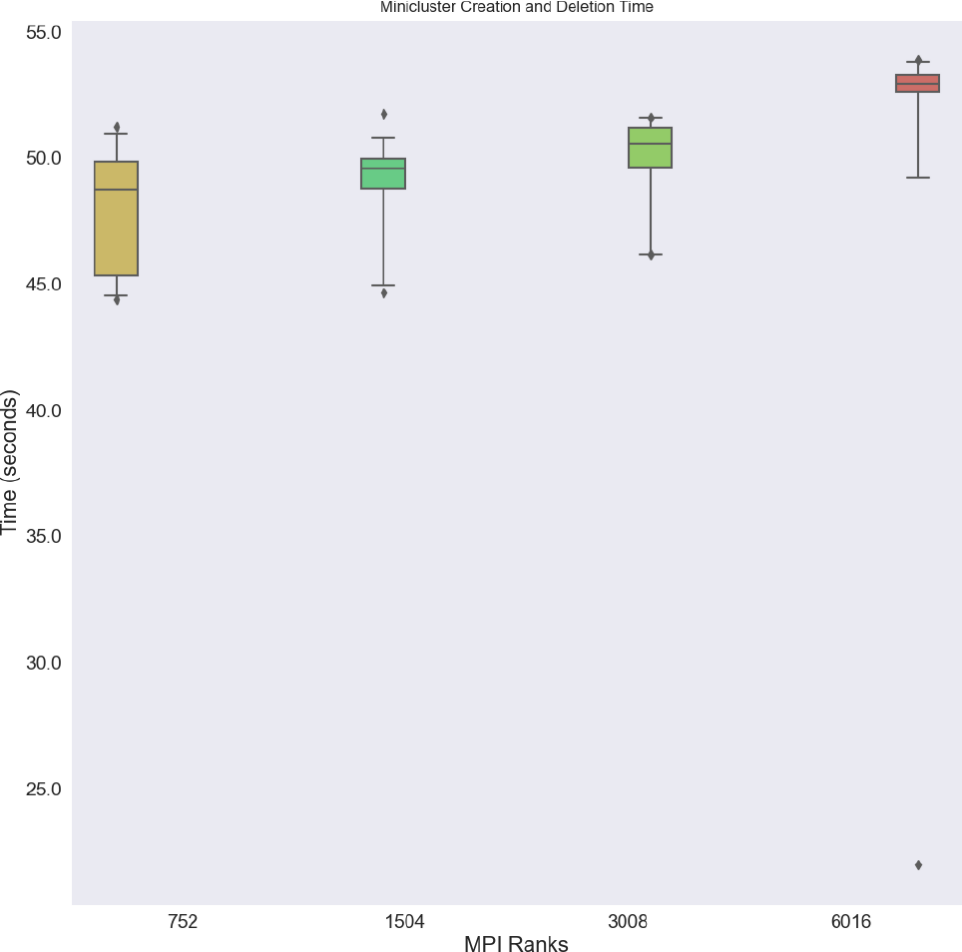
Combined Flux MiniCluster creation and deletion times across MPI ranks, corresponding to cluster sizes of 8, 16, 32, and 64 nodes, respectively. Times are calculated as the total time from creation to deletion, and subtraction of the time it took to run and job within. One outlier for size 6016 ranks stands out – one instance of the largest cluster completing creation, run, and deletion in a time closer to 50 seconds. These experiments were run on Amazon Web Services with the hpc6a.48xlarge instance type and the elastic fiber adapter for networking.

**Cluster creation and deletion** We created and deleted clusters of 8, 16, 32, and 64 nodes in 20 repetitions, observing a small increase in median times ( Figure 3) indicating that the indexed job can efficiently create pods. All sizes were created and ready in under a minute with an interquartile range for any given size between approximately 1 and 5 seconds, and a maximum difference of approximately 10 seconds between the fastest time of the smallest cluster and the slowest time of the largest cluster. Likely this variability reflects the slowest or last node to come up, as the cluster is not considered to be completely up until all nodes are ready. Due to the design of the MPI Operator, there was not a corresponding measure of creation time appropriate for comparison, so the two are not comparable side by side.

**Figure 4.**
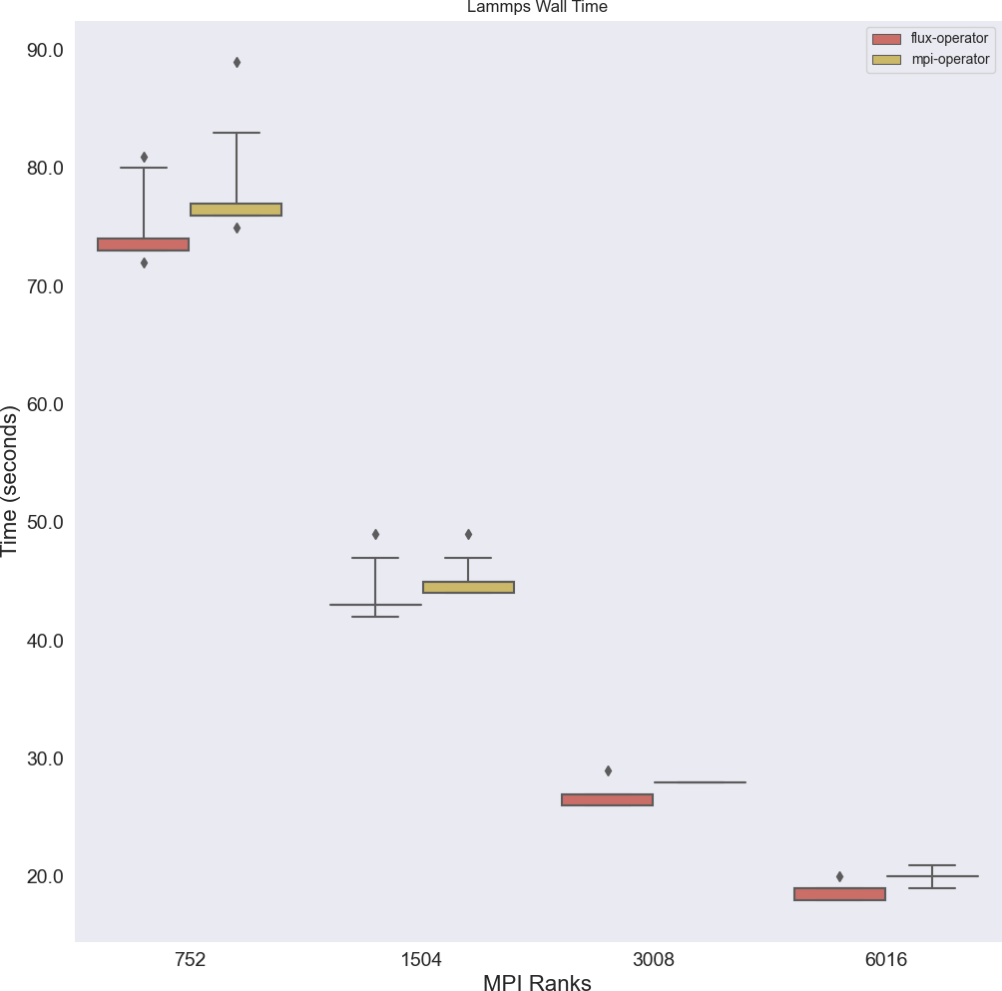
Total wall time, as reported by the LAMMPS software, between the Flux Operator and MPI Operator. The Flux Operator is consistently faster, a result that could be more impactful for longer running experiments.

**LAMMPS total wall time** A primary time of interest for running LAMMPS is the total wall time, which is reported by the LAMMPS software itself. Consistently better performance of the Flux Operator over the MPI Operator was observed, with median times that are approximately 5% faster, meaning that the Flux Operator software completed the same workload more efficiently ( Figure 4). While the differences in means for this experiment are small, this improvement would likely be more prominent for longer experiments, and could lead to reduction in total costs. Identifying the underlying reasons for the improved performance is another task suitable for investigation with performance tools in future work.

**Figure 5.**
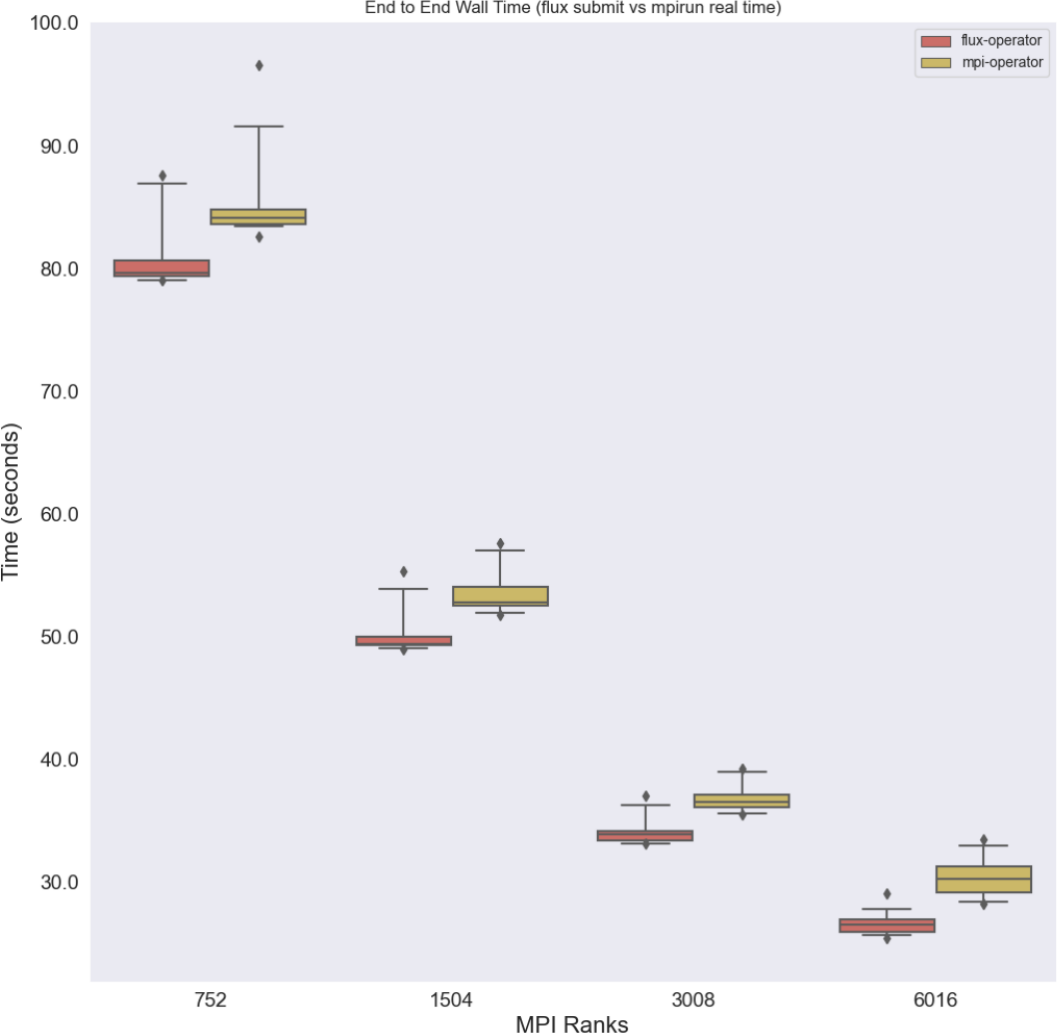
Launch time measuring flux submit (the Flux Operator) or mpirun (the MPI Operator). The Flux Operator is consistently faster, a result that could be more impactful for longer running experiments.

**Launcher times** Comparing launchers ( flux submit for the Flux Operator, and mpirun for the MPI Operator) there is a slightly larger time difference ( Figure 5), where both generally perform well under strong scaling (the time goes down). What is unknown is whether there is an inflection point at larger scales where the MPI Operator might plateau or otherwise show a different pattern. The resources were not available to run these larger experiments at the time, but these patterns and scaling can be explored in future work.

**Figure 6.**
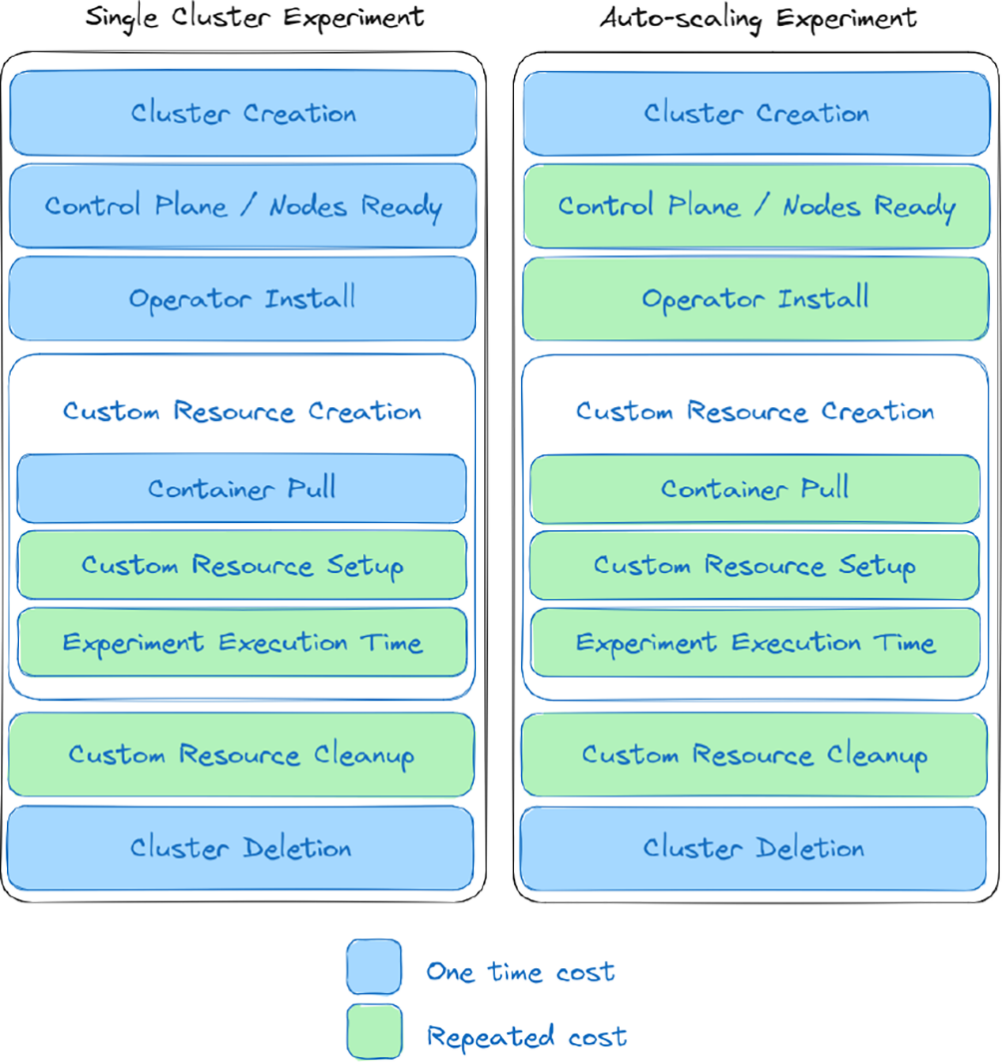
Times to consider when performing experiments with a Kubernetes operator, from the point of creating the cluster to deleting it. For a single cluster without autoscaling (left) many of the operations are one-time costs (blue), while for an autoscaling cluster that needs to provision new nodes and pull containers to them, the costs become repeated (green). An efficient approach might maximally use provisioned resources while waiting.

**Design considerations** Anticipating interest in running experiments of this type, where there is generally some operator in Kubernetes that is going to pull one or more containers to Kubernetes and perform scoped work, we provide a visualization of the steps that have salient times. In Figure 6, there is a distinction between an operator setup that is using autoscaling (right) vs. not (left), and costs that are incurred once (blue) vs. repeated (green). Cluster creation generally means the start and setup of instances, along with any networking and devices that are needed. The primary difference between the two scenarios is that an autoscaling cluster is going to be adding new nodes, meaning that the cluster will need to provision those nodes, and the user will be required to wait. Thus, this update process for the cluster that requires the user to wait (and pay for the time) becomes a repeated cost. This also means that a typically one-time cost of pulling a container may occur several times, primarily when new nodes are added. Note that this diagram assumes experiments running on one cluster. An autoscaling setup that employs bursting to new clusters would need to consider the additional time of creating and deleting the new clusters.

It is suggested to the reader to consider these times, along with the differences between a setup with autoscaling versus one without or potentially bursting, for future experiments when anticipating costs. Notably, the setup time for any particular operator could be generally consistent, and variance has been seen (but is not reported here) in the other steps between cloud providers. A more critical study and understanding of these times is warranted for future work.

## 4. Use cases

Once it was possible to run and complete a basic workflow (discussed in Section 3), development thinking moved toward adding desired use cases for such a workload manager in Kubernetes. This section will describe early work to enable scalability and saving state, elasticity and autoscaling along with workflow integration. These features for workflows, along with the core design of the Flux Operator, are considered experimental in the sense that they are implemented with the goal of testing and improvement in mind. The below represents a sample of this work, and more experiments can be found in the examples directory of the repository.

### 4.1 Saving state

The goal of experiments to save state would involve starting a Flux MiniCluster, running some number of jobs, pausing them, saving the state of the job queue, and then bringing it down to bring up a different sized cluster to load the jobs into, where they would continue running. This concept of saving state is similar to forensic container checkpointing in Kubernetes and checkpoint/restart (e.g., Ref. 31) in HPC, and would be useful for pausing workflows for cost savings or waiting on resource availability. These experiments varied based on when the queue was paused. In the earliest tests, job completion was required before saving state, while for later tests, jobs were stopped mid-run.

In practice saving state meant waiting for the queue, pausing, and then saving to an archive in a volume shared between two MiniClusters. Initially, the Operator waited for the first MiniCluster pods to terminate and the new MiniCluster to come up before restoring the jobs. We observed that jobs would successfully save and load into the new cluster, maintaining job identifiers and size, however when stopping a running queue, 1-2 jobs could be lost between the transfer. While the reason for this loss would be interesting to understand, as it is an experimental prototype for a feature, the work is beyond the scope of this paper, and akin to other features discussed here, should be pursued with a compelling research use case. When this time comes, more analysis would be needed to understand exactly what is going on. The majority of jobs (e.g., roughly of 9 out of 10) transition successfully, meaning that a job on the previous queue can get scheduled to a new larger or smaller cluster. As would be expected, if a job is moved onto a cluster lacking enough resources, it would logically not be scheduleable. A write-up and tutorial to reproduce this work is available.

While this early use case of saving state was simple, it was a glimpse into the idea that scheduled workflows could in fact be moved. In changing the size of the resources available by way of creating a new MiniCluster, it was the earliest prototype for what might be called scaling or elasticity, discussed next.

### 4.2 Elasticity

Elasticity can be thought of as automated dynamic scaling. ^32^ Instead of making a cluster larger or smaller by way of saving state and loading into a different size, true elasticity means changing the size of a single cluster, which in the context of the Flux Operator means that Flux must adapt dynamically. To enable MiniCluster elasticity we added limited support for resource dynamism within Flux. Our method is analogous to resource dynamism in Slurm in that it relies on configuring predefined resources. Support for resource dynamism by integrating arbitrary resources on demand is future work. The following steps enable an elastic Flux MiniCluster:

• A max size variable is added, meaning more nodes are defined in the system configuration file than actually exist.
• Flux is told to create a cluster at a size that is between 1 and this max size.
• Any change request to the CRD (from a user or API) validates the request, and updates the indexed Job.
• An update to increase in size creates new pods, and an update to decrease in size terminates pods.

The above also carries the constraints that the cluster cannot be smaller than one node (only a lead broker) or larger than the initial maxSize. The larger indices are terminated first and the operator does not allow reduction to size zero, so the lead broker is never at risk of deletion – such a request would delete the entire MiniCluster that relies on it. The reason this works is that Flux sees the initial set of pods that do not exist as simply being down, which happens frequently in a high performance computing environment. When the nodes are created their corresponding follower brokers start, ping the lead broker on the port to connect (typically port 8050) and then they seamlessly join the cluster. From the standpoint of the user, they change the “size” value in their MiniCluster CRD, apply it, and then see their cluster grow or shrink. The Flux instance run by the lead broker simply sees a node come online. On the Kubernetes side, this ability for the indexed Job to have elasticity requires a minimum Kubernetes version of 1.27.

At this point, it needed to be decided what might trigger this change in size. Elasticity was implemented in two ways, first with an application-driven approach that required extra permissions to be given to the in-cluster service account, allowing the application inside the cluster to ask for more or fewer pods directly. It was then discovered that Kubernetes has autoscaling APIs intended for this use case. This autoscaling approach is discussed in the next section.

### 4.3 Autoscaling

In Kubernetes there are two types of scaling – horizontal and vertical. Horizontal typically refers to adding pods, while vertical refers to adding resources to existing pods. Both are based on the idea that resources should change in response to changing workload needs – if cluster or resources are too big, they are made smaller, and vice versa. In the case of the Flux Operator the primary interest was horizontal autoscaling, or changing the number of pods to dynamically increase or decrease the size of the MiniCluster to respond to the demands of a workload. This led to an initial implementation based on horizontal pod autoscaling (HPA) using the HorizontalPodAutoscaler API resource, a cluster API to watch a selected set of pods for resource consumption and increase or decrease the number depending on utilization. For the simplest cases, a default autoscaler was first deployed that considers a metric such as percent CPU usage and uses an algorithm to calculate a target scale value for the number of pods. This could be tested by running a CPU intensive simulation to observe the autoscaler adding and removing pods. However, the approach was not fine-tuned enough to the potential needs of an application being run by Flux. Instead of an arbitrary decision to add or remove pods based on CPU, a design more specific to Flux is warranted. As an example, one design might be that the Flux lead broker makes decisions about when and how to scale depending on the content of the queue. Another valid design would be to allow for changing the size of a single running job. Both of these ideas, and more generally designs for autoscaling, are valid and prime for future work.

With this in mind, we implemented a custom metrics API, meaning implementing an equivalent API endpoint controller that would be called by an autoscaler with instructions for how to scale the cluster. This resulted in the Flux metrics API, a standalone API that runs directly from the lead broker pod and provides decisions about scaling up or down based on the size or other metrics about the queue. With this API, it was possible to demonstrate an autoscaling operation running based on a trigger coming directly from Flux. More work will be needed to test this setup with real workflows. In the meantime, more details about this setup and basic elasticity are available in an external post.

One notable feature about the implementation of the autoscaling approaches described above is that regardless of whether the request comes directly from a user changing a value in a file or an application or a programmatic autoscaler, the same internal logic (functions) is used to validate and then perform the patch.

### 4.4 Multi-tenancy

Multi-tenancy refers to the ability to support multiple users on the same resources. In Kubernetes, ownership of resources is typically designated by namespaces, service accounts, custom permissions on connected resources like storage, and role based access controls (RBAC). Recognizing these challenges, as an early approach there are several modes of interaction:

• Single user: the user owns an entire MiniCluster, and uses the default Flux user in the container
• Multiple users: controlled via PAM authentication
• Multiple users: controlled via RESTFul API access

In anticipation of the last two cases that implement multi-tenancy, a RESTful application programming interface (API) was designed that runs from the lead Flux broker pod, and thus exposes interactions with Flux to submit, get info, cancel, and otherwise interact with jobs via Flux Python bindings exposed by the API. This is made possible by exposing the internal port that the API is running on via an external NodePort and forwarding a port to an external client for interaction.

In all cases of requiring authentication, the Flux RESTful API uses an OAuth2 based approach, storing a database of user identifiers and encoded passwords, and first authenticates by using a base64 encoded username and password (typical of a basic authentication scheme), and then provides the user with an expiring token that can be used for further interaction. In the case of a single Flux user behind a multi-tenant API, the authentication and authorization occurs, allowing all users to submit jobs to a shared queue. In the case of true multi-tenancy with PAM, the custom resource definition asks for usernames (and optionally, passwords) in advance, and then creates the accounts on the system that are checked after authentication. The installation of flux-accounting can then be enabled for the lead broker’s queue, and use a traditional fair-share algorithm to determine job priority. This work can be extended with more cloud-native approaches that take advantage of namespaces and roles, such as is described later ( Section 4.6).

### 4.5 Bursting

To complete the early work in autoscaling, we considered the concept of bursting, ^33^ which means not just extending the size of a local cluster, but actually extending work to external resources. The bursting work for Flux would extend this approach to not just deploy external resources, but allow the lead broker to connect to brokers that are deployed in the other clusters. As an example, a Kubernetes cluster running on Google Cloud might burst to a cluster running on Compute Engine (CE), or to a cluster on Amazon Elastic Kubernetes Service (EKS).

To implement a prototype for bursting, we chose a simple design first. A plugin service would be running from the lead broker of a primary cluster, and the running user would load one or more bursting plugins into it. Each bursting plugin is targeted to a particular provider (e.g., EC2 or CE). While there are many ways to trigger a burst, a simple approach of looking for an attribute “burstable” on a job set to true was chosen first. This request could be done on the command line. Upon discovery of this attribute, the bursting service attempts to schedule the job with the plugin. Each plugin is free to decide if the request is satisfiable by its own custom terms. If the burst is satisfiable, the job is assigned to the bursting plugin, and the plugin creates a new cluster or assign the job to an existing cluster. In the case of creation, the technique of telling the primary cluster that there are more nodes that are expected (and start down) than there actually are is used, and assign them namespaced hostnames that will correspond to the bursted cluster. The calls that are necessary to bring up the second cluster are run, which might mean deploying Terraform configuration files or creating a second Kubernetes cluster via API calls, and then the cluster starts just as a local MiniCluster would. The key difference, however, is that the lead broker of the primary cluster is exposed as a NodePort service that can be discovered by the external cluster. The secondary brokers, all followers, then come up, find their hostnames in the ranked system configuration, and connect to the lead broker IP address from another cluster. To the user, they simply see that the nodes are down, and then they come up when the cluster is ready. Jobs that are scheduled on the primary broker queue that possibly could not run due to insufficient resources can then run. At the time of this writing, the main bursting service is implemented along with four bursting plugins for each of GKE, EKS, CE, and a local burst.

Finally, the bursting service is designed to be modular and flexible. Aside from being able to load different plugins, it allows for customization of the function provided to select a burstable plugin, to interact with the queue, and to select jobs. A mock version of a Flux job is also available for development. The work in bursting is still early, and akin to elasticity, work on these prototypes should continue to eventually develop more hardened Flux modules and algorithms for bursting.

### 4.6 Workflow integration

Running a single MiniCluster to create an isolated Flux cluster in Kubernetes is an initial step, but it is insufficient for real-world use cases of complex workflows. While it would be possible to shell into or otherwise interact with the cluster and run a workflow tool that implements Flux as an executor, ^34^^–^^36^ this also does not enable features needed for complex, heterogeneous workflows that might require different sizes or configurations of MiniClusters. We are considering integration of the MiniCluster custom resource definition as a first-class citizen into workflow tools in future work.

After the Kubecon Amsterdam’23 presentation, collaborators (including author AC) were quickly motivated to add the Flux Operator as a job type to the workflow tool Kueue, a Kubernetes-native job submission operator that handles managing multi-tenancy of Kubernetes batch jobs that can be produced by a number of operators. We are developing a similar approach to control the creation and management of a MiniCluster through workflow tools, an idea being implemented into the Snakemake ^34^ workflow tool as a Kueue executor plugin. Defining even an example workflow for high performance computing is a non-trivial problem, as many codes are either private or challenging to port. This initial work with the Flux Operator sets the stage for studies of integrations of this type. Tackling this early problem is a two-fold challenge to design technologies and inspire more collaborative opportunities for the HPC community.

## 5. Discussion

This work demonstrates improved performance using the Flux Operator for running an HPC workflow in a cloud-native environment. This early innovation comes with strengths, limitations, and identification of important future work that includes workloads and scheduling, storage, tenancy, and cost-estimation, among others.

A discussion of limitations and further hopes for innovation is needed for transparency of this work. First, it is a design flaw that the main execution container is required to have Flux and the application of interest. This means that any user of the Flux Operator is required to rebuild their container in full to include Flux, which requires knowledge of Flux, and in the case of using the message passing interface (MPI) knowledge of MPI. While container requirements are provided alongside the documentation, a better approach would not place such requirements on the application container. While the dependencies and complexity exist to enable advanced capabilities, the authors believe there are approaches that can improve upon this strict requirement.

A next limitation is the creation of the entire MiniCluster using a single indexed Job. While this is the ideal for the time being, as the indexed Job is released with core Kubernetes, an eventual refactor to use a JobSet would be desired, which can create multiple Indexed Jobs underneath, however allowing for different configurations for the lead and follower brokers, and an ability to define different groups of nodes each as a Replicated Job under the same network namespace. Allowing for different sets of nodes would not only make it possible to separate logic between the lead and follower brokers, but also allow for creation of a MiniCluster with different pod specifications mapped to different resource needs. JobSet would also allow for better definition of a success policy, or explicitly reporting job completion when the lead broker exits. Author VS created a prototype using JobSet, anticipating its integration in Kubernetes core.

For next steps of work for experimental features, the Flux software itself needs innovation for the set of the hacks that were implemented. The ability to scale up and down dynamically without “registering” the non-existent nodes in the Flux system configuration is a good example, along with a more hardened approach for bursting that likely comes down to plugins written directly in C or C++ alongside Flux. Especially the work in bursting is early and exciting, and will be continued in future work. Notably, the experiment application did not require use of storage, and while there are several tutorials and examples for different cloud solutions, this is an entire area of the design that requires further work and thinking.

Another challenge is that of poor workflow consistency and reproducibility. While there are scattered workflow tools that are used by HPC centers (e.g., national labs), these authors consider much of the HPC community behind with respect to the reproducible workflow movement. Part of this work moving forward is to not only identify proxy applications and workflows, but also to containerize them, and make it quick for an interested party to run them easily in a cloud environment. Part of this work will not only be understanding how they work in containers and across a Kubernetes cluster, but also developing means to assess performance.

An understated challenge in the converged computing space is also culture and communication. As stated in the introduction, convincing one side to be open to ideas from the other is a non-trivial task. For basic communication, if there is discussion between HPC and cloud community members, a simple term like “node” or “scheduler” can mean something different. This might be tackled through discussion, and creation of a shared lexicon that allows for talking about comparable distractions. This introduces a further challenge when looking at the means for communication. Academic groups tend to write papers (and industry groups less so), and developing software in research is made more complex by the publication incentive structure that wants to highlight new research results. ^37^ Practically speaking, both the HPC and cloud communities will need to meet one another half way. This might mean researchers presenting work at (traditionally) more cloud-oriented conferences and venues, or cloud developers participating in more traditionally research-oriented venues. For both, it means distributing knowledge through blogs and other common mediums. This work calls out to cloud vendors an immense desire to work together. While it is understandable that there is a primary concern about direct comparison, there is a path for respectful collaboration, developing technologies that can work across clouds, and learning from one another.

These experiments were run on one cloud with a particular networking fabric and instance type, and at a maximum scale of 64 nodes, which is very small for traditional HPC. However, the recent work to train large language models ^38^ provides a common use case for needing scaled resources, and might allow for shifting incentives toward that. One of the most challenging decision points for running experiments of this nature is the sheer size of the space of potential experiments that could be run. As the goal of this work was to compare the two operators with an HPC application, the choices made reflect that goal, and a desire to optimize performance (choosing a configuration to support low network latency) as much as possible. These same experiments run on other instance types, interconnects, or even regions could have different results. Further experiments should be pursued that continue to test scale, elasticity, and performance in the space of networking, I/O, and application metrics.

The Flux Operator brings several features that could be helpful to more general Kubernetes workflows. The first is that using a Flux cluster inside of Kubernetes gets around some of the infamous etcd limits or bottlenecks. ^39^ Submitting to Flux does not stress Kubernetes application programming interfaces or etcd, and could scale to hundreds of thousands to potentially millions of jobs. ^16^ The second is the hierarchical way of looking at heterogeneous tasks. Kubernetes would benefit from having more flexibility about telling tasks where they can go, and then binding them to exactly the resources needed. This brings up tension between a more manual vs. automated decision made by the Kubernetes kubelet. The Flux Operator does something that is not native to Kubernetes to help this issue. By way of allocating a pod to a node and giving control to Flux, possibly ineffective bindings decisions that are made by the kubelet can be avoided. The Flux Operator allows Flux, a workload manager accustomed to making intelligent resource bindings, to take control. Ideally there should (or could) be a mechanism in Kubernetes to enable more performance oriented decisions that the kubelet makes. Having a consistent view of resources that the Kubelet is exporting as the final truth via cgroups is not necessarily desirable, and there are several reasons why. The first is that there are many ways to slice up a node, and a “best” way depends on the application in question. For example, some applications may perform well given an equal split, while others might be optimally be broken across sockets. This suggests that granularity on the level of the socket and below is needed, which is not currently exposed in Kubernetes, but being worked on. A concrete example comes from the MuMMI workflow ^40^^,^^41^ that requires a task to be bound to CPU cores closest to a PCI express bus to minimize communication latency with a GPU. This level of granularity is not currently exposed in Kubernetes, and arguably there need to be more consolidated efforts to understand applications on this level. This is a key area for innovation and collaboration, and understanding basic design patterns for networking, I/O, and application performance is likely a good start. Ideally applications that are running in Kubernetes today could be better understood via performance analysis, and a decision made about if the time required to optimize is worth to be invested for the potential benefit.

## 6. Conclusions

The popularity and economic clout behind cloud computing presents a challenge for the high performance computing community – to resist new paradigms of cloud-native technologies and fall behind, losing talent and customers, or to embrace it, pursuing technological innovation, and viewing the shift as an opportunity for collaboration and growth. The latter approach, a movement identified as “converged computing” is a good path to take, and this work represents an early start towards that desired future. The work here starts with one of the highest levels for running workflows – the orchestration tool – and has thought about the convergence of the HPC workload manager Flux Framework with the cloud orchestration framework Kubernetes. The Flux Operator is an example of convergence of technologies in this workload management space, and has demonstrated superior performance to the current equivalent in the space. In sharing this process of thinking about design to implementation, the authors of this paper hope to start discussion with the larger community that spans cloud and HPC for how to think about working on convergence for other types of software and technology. This work is a shining example of the collaboration and fun possible. The sharing of these results at Kubecon Amsterdam’23, ^23^ inspired collaboration, discussion, and excitement about the converged computing movement. Projects and work are underway to address gaps that have been identified, and each a collaboration between computer scientists and cloud developers. The authors of this paper hope that this early work inspires, and allows for continued discussion for innovation in this space for not just workloads and scheduling, but also the challenges around it – storage, tenancy, and cost-estimation, among others. Through collaboration and creativity of design, pathways can be discovered for moving seamlessly between the spaces of cloud and HPC. Converged computing is a paradigm shift, and it’s up to the HPC and cloud communtities to decide to embrace change and grow from it, or fight it off. This work chooses the first approach, and embraces it with hopes for a better future, and stronger more reproducible science.

## Data Availability

Zenodo: Underlying data for’The Flux Operator’, converged-computing/operator-experiments: F1000Research submission release, https://doi.org/10.5281/zenodo.10248093. ^29^ This archive contains the following underlying data:
• *.yaml: configuration files • results: experimental data Data are available under the terms of the Creative Commons Attribution 4.0 International license (CC-BY 4.0).

## Software availability

Source code available from: The Flux Operator: https://github.com/flux-framework/flux-operator/releases/tag/0.1.1

Archived source code at time of publication: Zenodo: 10.5281/zenodo.8312253. ^30^

License: MIT License
