# Supplementary material for: The Flux Operator
Source: F1000Res. 2024 Mar 21;13:203. [Version 1] doi: 10.12688/f1000research.147989.1 (PMC11167326; doi:10.12688/f1000research.147989.1)
Supplement: Peer review history

# Reviewer report: Reviewer response for version 1

**DOI:** 10.5256/f1000research.162246.r284364

**Published:** 2024-06-11

**Authors:** David Hancock

**Affiliations:** 1 Pervasive Technology Institute, Indiana University, Bloomington, USA

This paper presents a comparison of two operators to execute HPC workflows within Kubernetes. It provides detailed background and motivations for using Flux as well as motivations for "converged computing" in general. Ultimately it aims to show better efficiency at modest scale of the Flux Operator over the MPI Operators for LAMMPS

 Some minor tweaks would improve readability as well as ideas for future work. 

 Language in the Abstract: Referring to Kubernetes as a "batch workload orchestrator" seems odd, certainly a de facto standard but most approaching Kubernetes would not be approaching it from a batch mindset. It is certainly tied together later on when speaking about Kueue but I think the audience from either HPC or Cloud might balk at that descriptor. 

 Language in the in Introduction: "While a traditional HPC job is, e.g..." This sentence with an abbreviated example given embedded makes it very difficult to read/parse, consider... As an example a traditional HPC job is/may be/can be...

 The data in Table 1 are concise enough that a table seems unnecessary at all and either a text-based description or just pointer to the guide would suffice. It does not seem to contribute to the overall article much. 

 The conclusion is quite philosophical and well-beyond the original stated goal. Perhaps a better way to start it is to wrap up the technical conclusion based on the original premise and then use the remainder to move into that area or add another section on motivations in/after the discussion. I don't disagree with many of those thoughts they just seem a bit out of sync with the specific goal. 

 Another curiosity or area of exploration in other work would be to look at this challenge from a user/operator perspective, what is the uptake among the current user base  from an HPC center and certainly there are LLNL plans to use Flux at scale what interest is being seen elsewhere? Conversely understanding uptake of the MPI Operator from a Cloud perspective would be interesting if such data are available. These are perhaps out of scope for this article but questions many readers may have with respect to Flux.

Are the conclusions about the tool and its performance adequately supported by the findings presented in the article?

Yes

Is the rationale for developing the new software tool clearly explained?

Yes

Is the description of the software tool technically sound?

Yes

Are sufficient details of the code, methods and analysis (if applicable) provided to allow replication of the software development and its use by others?

Yes

Is sufficient information provided to allow interpretation of the expected output datasets and any results generated using the tool?

Yes

Reviewer Expertise:

return on investment of research computing, high performance computing and cloud systems architecture and deployment.

I confirm that I have read this submission and believe that I have an appropriate level of expertise to confirm that it is of an acceptable scientific standard.

# Reviewer report: Reviewer response for version 1

**DOI:** 10.5256/f1000research.162246.r267225

**Published:** 2024-06-07

**Authors:** Rafael Ferreira da Silva

**Affiliations:** 1 National Center for Computational Sciences, Oak Ridge National Laboratory, Oak Ridge, Tennessee, USA

This paper introduces an approach to converged computing by integrating the Flux Framework within Kubernetes. This integration aims to leverage the benefits of both HPC and cloud-native environments to improve workflow portability, flexibility, and manageability. The Flux Operator enables hierarchical resource management and scheduling within Kubernetes, addressing the growing need for efficient and scalable batch workload orchestration. This paper details the design decisions, component mappings, and experimental features of the Flux Operator, highlighting its ability to enhance performance compared to existing solutions like the MPI Operator. Experiments conducted using AWS Elastic Kubernetes Service (EKS) demonstrates improved performance of the Flux Operator in running HPC applications such as LAMMPS.

 Minor comments for improvements:

 - The definition of modern workflows in the introduction should be broadened to include AI-HPC workflows. These workflows are characterized by their dynamic behaviors, human-in-the-loop processes, conditionals, and loops. Incorporating this definition will provide a more comprehensive overview of the current landscape of HPC workflows and highlight the increasing complexity and versatility of these tasks.

 - In Section 2.1.3, it would be beneficial to provide insights on potential approaches for achieving separation of application logic from Flux without impacting performance. This could include exploring containerization strategies that decouple the application and Flux components, using lightweight virtualization techniques, or employing modular software design principles that isolate application-specific logic from the underlying workload management framework.

 - The experimental evaluation in Section 3.1 would benefit from including an experiment that compares the overall time-to-solution for running an experiment using the Flux Operator versus the MPI Operator. Specifically, it would be useful to analyze the overhead or cost associated with preparing the environment for the operator (e.g., pulling the container) and the subsequent performance gains over time compared to the MPI Operator.

 - It would be helpful to briefly discuss the cause of the outlier observed for 6016 ranks in Figure 3.

 - In Section 3.2, the variability shown in Figure 3 would be better expressed if a distribution of nodes-ready times (per node) were included. Presenting this data would offer a more granular view of the time it takes for individual nodes to become ready, highlighting any significant deviations and providing insights into the factors influencing node startup times.

Are the conclusions about the tool and its performance adequately supported by the findings presented in the article?

Yes

Is the rationale for developing the new software tool clearly explained?

Yes

Is the description of the software tool technically sound?

Yes

Are sufficient details of the code, methods and analysis (if applicable) provided to allow replication of the software development and its use by others?

Yes

Is sufficient information provided to allow interpretation of the expected output datasets and any results generated using the tool?

Yes

Reviewer Expertise:

Distributed computing; scientific workflows; high performance computing

I confirm that I have read this submission and believe that I have an appropriate level of expertise to confirm that it is of an acceptable scientific standard.
